# Extended Spectrum Beta‐Lactamase Production and Multidrug‐Resistant Enterobacterales Among Patients With Symptoms of Urinary Tract Infection in Addis Ababa, Ethiopia

**DOI:** 10.1155/bmri/7993514

**Published:** 2026-08-21

**Authors:** Tamirat Tamiru, Yordanos Getachew, Sami Sebri, Regasa Diriba, Mekdelawit Fisseha, Tadesse Shume, Kassu Desta

**Affiliations:** ^1^ Department of Microbiology Laboratory, Wudassie Advanced Medical Laboratory, Addis Ababa, Ethiopia; ^2^ Department of Vaccine, Diagnostic and Medical Device Research and Development, Armauer Hansen Research Institute, Addis Ababa, Ethiopia, ahri.gov.et; ^3^ Department of Medical Laboratory Sciences, College of Health Sciences, Addis Ababa University, Addis Ababa, Ethiopia, aau.edu.et

**Keywords:** Enterobacterales, Ethiopia, extended spectrum *β*-lactamases, multidrug resistance, UTI

## Abstract

**Background:**

Urinary tract infections (UTIs) are one of the commonly encountered infectious diseases globally, affecting approximately 150 million individuals each year. The rise of multidrug‐resistant (MDR) particularly extended spectrum beta‐lactamase (ESBL) producing Enterobacterales has made the management of UTI more difficult and this is becoming a common public health concern worldwide. The study is aimed at assessing the prevalence, antimicrobial resistance, and extended‐spectrum beta‐lactamase–producing Enterobacterales among patients with symptoms of UTI at Wudassie Advanced Medical Laboratory, Addis Ababa, Ethiopia.

**Methods:**

A laboratory based cross‐sectional study was conducted from April to July 2025. A total of 502 urine specimens from UTI suspected patients were included in the study. The identification of Enterobacterales was done using MALDI‐TOF MS. Antimicrobial susceptibility testing was performed using the Kirby–Bauer disc diffusion technique. Combined disc test (CDT) was used for confirmation of ESBL producing Enterobacterales. Data was entered in Epidata version 4.6 and analyzed using SPSS Version 27.

**Result:**

Among 502 patients presenting with clinical symptoms of UTI, 29.4% (148/502) had positive culture for Enterobacterales. The predominant species isolated were *Escherichia coli* 103 (69.6%) and *Klebsiella pneumoniae* 25 (16.8%). The prevalence of extended spectrum *β*‐lactamases–producing Enterobacterales (ESBLs‐E) was 65.5% (97/148: 95% CI: 57.8%‐73.2%). The highest proportion (92.3%) of ESBL‐producing isolate among inpatients was *K. pneumoniae*. MDR was also detected in 82.4% of the isolates from this study.

**Conclusion:**

The findings highlight a high prevalence of MDR‐ and ESBL‐producing Enterobacterales causing UTI in Ethiopia. This finding emphasizes a critical need to implement comprehensive surveillance systems in hospital settings to enable the detection and tracking of MDR Enterobacterales. Future research needs to be conducted to direct policies on the implementation of antibiotic stewardship programs.

## 1. Introduction

Urinary tract infections (UTIs) are among the most common infectious diseases worldwide, affecting approximately 150 million people annually and imposing a substantial burden on both community and hospital settings [1]. It accounts for 35% of nosocomial infections and approximately 1 million hospitalizations worldwide [2]. This translates to about 40% of women and 12% of men experiencing at least one symptomatic infection in a lifetime. The increasing prevalence of multidrug resistance (MDR) and extended spectrum *β*‐lactamase–producing Enterobacterales (ESBL‐E) poses a significant threat to the effective management of UTIs [3].

Many classes of antibiotics are used to treat UTIs, among them the beta‐lactam antibiotics are the most commonly used medications in clinical practice because of their better safety profiles and broad‐spectrum activities [4]. Numerous studies have indicated that the emergence of antibiotic resistance is increasing due to the practice of self‐medication and uncontrolled use of antibiotic prophylaxis resulting in selection pressure on the bacterial populations [5].

UTI caused by antibiotic resistant Enterobacterales makes empirical treatment of these infections difficult. These groups of bacteria employ multiple resistance mechanisms, including the production of enzymes such as ESBLs, AmpC *β*‐lactamases, and carbapenemases [6]. ESBLs hydrolyze and inactivate beta‐lactam antibiotics, rendering them ineffective against infections caused by ESBL‐producing bacteria. ESBL‐E are resistant to penicillins, third‐generation cephalosporins, and monobactams (aztreonam), but not to cephamycins (cefoxitin and cefotetan) and carbapenems [7]. However, they are susceptible to *β*‐lactamase inhibitors such as clavulanic acid, sulbactam, and tazobactam [8].

Among Enterobacterales, ESBLs have been commonly observed in *Klebsiella* and *Escherichia coli* spp., though it has also been reported in other genera of Enterobacterales such as *Proteus* spp.*, Enterobacter* spp.*, Citrobacter* spp.*, Serratia* spp.*, Morganella* spp.*, Providencia* spp. and *Salmonella* spp. [9]. The global rise in ESBL‐E has contributed significantly to MDR in both community and hospital settings. ESBL‐producing *Klebsiella pneumoniae and E. coli* have become a major global public health threat [10].

Thus, many ESBL‐producing bacteria frequently exhibit resistance to multiple non–*β*‐lactam antibiotic classes, such as quinolones, tetracyclines, sulfonamides, aminoglycosides, trimethoprim‐sulfamethoxazole, and chloramphenicol, which are commonly used in treating severe bacterial infections [11–13].

The phenomenon of antimicrobial resistance among ESBL‐E isolates of various samples has been reported in Ethiopia [9, 14]. However there has not been a well described profile of ESBL‐E isolates from UTI patients especially from those visiting private health facilities. Thus, this study tried to describe the bacterial profiles, their antimicrobial susceptibility, and the prevalence of MDR‐ and ESBL‐E among urine samples referred to a stand‐alone diagnostic laboratory from different health facilities of Addis Ababa, Ethiopia. This study was designed to assess extended spectrum beta‐lactamase (ESBL) production and MDR Enterobacterales among UTI‐suspected patients referred to Wudassie Advanced Medical Laboratory (WAML), Addis Ababa, Ethiopia.

## 2. Materials and Methods

### 2.1. Study Settings

A laboratory‐based cross‐sectional study was conducted from April to July 2025 at WAML, Addis Ababa, Ethiopia. WAML is a private, ISO 15189–accredited diagnostic laboratory that receives specimens from hospitals, health centers, and clinics across the city. The laboratory provides routine bacteriology services, including culture, antimicrobial susceptibility testing (AST), and matrix‐assisted laser desorption/ionization time‐of‐flight mass spectrometry (MALDI‐TOF MS) identification.

## 3. Population

### 3.1. Source Population

All UTI suspected patients referred to WAML for urine culture during the study period were the source population for this study.

### 3.2. Study Population

The study population were patients clinically suspected of UTI, who meet the eligibility criteria, and volunteer to participate.

### 3.3. Inclusion Criteria

Patients of any age and sex and who were clinically suspected of UTI were included as part of the criteria.

### 3.4. Exclusion Criteria

Patients who had taken antibiotics within 7 days prior to specimen collection were excluded.

In this study, profiles of Enterobacterales, antimicrobial susceptibility patterns of Enterobacterales, and prevalence of ESBL‐E were our dependent variables. Age, sex, health facility type, and patient department were an independent variable.

## 4. Sample Size Calculation and Sampling Method

### 4.1. Sample Size Calculation

The sample size was calculated using the single population proportion formula based on the prevalence of ESBL‐E among patients clinically suspected of UTI. A prevalence (p) of 61.7% (0.617), reported in a previous study conducted at Tikur Anbessa Specialized Teaching Hospital, Addis Ababa, Ethiopia [15], was used, with a 95% confidence level (*Z* = 1.96) and a margin of error of 5% (*d* = 0.05). The minimum required sample size was 363 participants. To improve the representativeness of the study population and enhance the generalizability of the findings, a total of 502 eligible patients with clinically suspected UTI were consecutively enrolled during the study period. Following urine culture and bacterial identification, only participants with culture‐confirmed Enterobacterales isolates (*n* = 148) were included in the subsequent analyses of ESBL production and MDR.

### 4.2. Sampling Method

A Cross‐sectional study design was used and participants were recruited using convenience sampling method.

### 4.3. Data Collection Procedure

Trained laboratory personnel, who took training from the principal investigator, employed a prestructured laboratory data collection form to gather sociodemographic data from patients.

## 5. Laboratory Analysis

### 5.1. Urine Specimen Collection, Handling, and Transport

A clean catch morning midstream urine specimen (20 mL) was collected from patients suspected of UTI using a sterile screw‐capped, wide‐mouth container and labeled with the unique sample number, date, and time of collection. After collection, the specimens were transported immediately to the microbiology laboratory at WAML, using an icebox, and stored at 2°C–8°C if testing is delayed beyond 2 h.

### 5.2. Culture and Bacterial Identification

About 1 uL of urine specimen was streaked on MacConkey Agar (Oxoid, Basingstoke, United Kingdom) and 5% sheep Blood agar (Oxoid, Basingstoke, United Kingdom) and then incubated at 37°C for 24 h [16]. Plates were observed for the appearance of characteristic colonies and bacterial growths of ≥ 105 CFU/mL were considered significant bacteriuria. All isolates were identified using MALDI‐TOF MS (EXS3000, Zybio Inc., China) [17].

### 5.3. Antibiotic Susceptibility Testing

Antimicrobial susceptibility was done by using the Kirby–Bauer disc diffusion method and categorized as susceptible, intermediate or resistant according to CLSI 2024 guideline. Bacterial suspension adjusted to 0.5 McFarland turbidity inoculums was prepared and inoculated on Muller–Hinton Agar (MHA) plates (Oxoid LTD, Basingstoke, Hampshire, England) and antimicrobial discs were applied to the plate. The antibiotic discs used in this study were ampicillin (AM: 10 *μ*g), amoxicillin‐clavulanic acid (AMC: 20/10 *μ*g), ceftazidime (CAZ: 30 *μ*g), cefotaxime (CTX: 30 *μ*g), cefepime (FEP: 30 *μ*g), gentamicin (GEN: 10 *μ*g), amikacin (30 *μ*g), nitrofurantoin (NI: 300 *μ*g), and sulfamethoxazole‐trimethoprim (SXT: 3.75/1.25 *μ*g), ciprofloxacin (CIP: 5 *μ*g), meropenem (MER: 10 *μ*g).

### 5.4. Multiple Antibiotic Resistance (MAR) Index Determination

The MAR index was determined for each Enterobacterales isolate to assess the extent of antimicrobial resistance and the likely level of antibiotic selective pressure in the source environment. The MAR index was calculated using the formula MAR = *a*/*b*, where *a* represented the number of antibiotics to which an isolate was resistant, and *b* represented the total number of antibiotics tested against that isolate.

The MAR index values ranged from 0 to 1, with higher values indicating greater resistance to the tested antimicrobial agents. Isolates with a MAR index greater than 0.20 were considered to have originated from environments with frequent exposure to antimicrobial agents or high‐risk sources, where antibiotics are commonly used. Conversely, isolates with a MAR index of 0.20 or less were considered to originate from environments with lower antibiotic selective pressure.

### 5.5. Detection and Screening of ESBL Production

ESBL production of Enterobacterales was screened considering the zone of inhibition diameters produced by cefotaxime (30 *μ*g) or ceftazidime (30 *μ*g) in the disc diffusion technique. Enterobacterales isolates with zone of inhibition diameters of ≤ 22 mm for ceftazidime and ≤ 27 mm for cefotaxime were considered as likely ESBL producers according to the CLSI 2024 [18]. Enterobacterales isolates that tested positive during ESBL screening were subcultured onto 5% sheep blood agar to obtain fresh bacterial colonies for phenotypic confirmation of ESBL production. The purified isolates were then preserved in tryptic soy broth supplemented with 20% glycerol and stored at −80°C.

### 5.6. Confirmation of ESBL Production

ESBL production among potential ESBL‐producing isolates was confirmed phenotypically using combined disc test (CDT). Comparison of the zone of inhibition was made for the cefotaxime (30 *μ*g) and ceftazidime (30 *μ*g) discs alone versus that of the cefotaxime‐clavulanic acid (30 *μ*g/10 *μ*g) and ceftazidime (30 *μ*g)‐clavulanic acid (30 *μ*g/10 *μ*g) placed at appropriate distance on MHA plate inoculated with a bacterial suspension of 0.5 McFarland turbidity standards and incubated overnight (18–24 h) at 37°C. An increase in the inhibition zone diameter of ≥ 5 mm for a combination disc versus ceftazidime or cefotaxime disc alone was confirmed as ESBLs production as per CLSI 2024 guideline [18].

### 5.7. Data Quality Assurance

To ensure data quality, the data collection form was pretested and the data that has been collected was thoroughly checked on spot and daily for their completeness, accuracy, and clarity. All of the preanalytical, analytical and postanalytical steps were carried out in strict adherence to the standard operating procedures (SOPs) of the microbiology laboratory at WAML to assure the quality of laboratory results. All urine culture specimens were collected by well‐trained laboratory personnel following the SOP. The expiry date of media, antibiotic disc and reagents, as well as the sterility and performance of media and antibiotics, were checked by known standard strains of *E. coli ATCC 25922*. Proper aseptic techniques were used when collecting and handling urine samples. Sterility of the culture media was checked with indicator tape and by incubating 3%–5% of the batch at 37°C for 24 h. Media performance was further evaluated by inoculating with control strains. Calibration of MALDI‐TOF MS was performed annually according to manufacturer instructions. The reference bacterial strains such as *K. pneumoniae* ATCC 700603 and *E. coli* ATCC 25922 were used as a control in each batch of microbial culture. To normalize the density of inoculums of bacterial suspension for susceptibility testing, 0.5 McFarland standards were used. Reporting of the processed urine specimen adhered to a standardized format and terminology to ensure consistency and clarity.

### 5.8. Data Analysis and Interpretation

Data were entered using Epidata Version 4.6 and analyzed using SPSS software (Version 27). Descriptive statistics including frequencies, percentages, tables, and figures were used to summarize demographic characteristics, bacterial profiles, ESBL prevalence, and antimicrobial resistance patterns. The dependent variables in this study were the profile of Enterobacterales, the antimicrobial susceptibility patterns of Enterobacterales, and the prevalence of ESBL‐E. The independent variables included age, sex, health facility type, and patient department. Cross‐tabulations were used to describe the distribution of the outcome variables across these independent categories.

### 5.9. Operational Definitions

Operational terms are defined as follows:

• Suspected UTI patient: Patients presenting with one or more urinary symptoms (such as dysuria, urinary frequency, urgency, or suprapubic pain) [19].
• ESBL: Enterobacterales isolates demonstrating synergy between ceftazidime or cefotaxime and clavulanic acid using the CDT, following the Clinical and Laboratory Standards Institute guidelines Version 24 [18].
• MDR: Isolates exhibiting nonsusceptibility to at least one agent in three or more antimicrobial classes [20].
• Enterobacterales: gram‐negative, rod‐shaped, facultative anaerobic bacteria, including clinically relevant genera such as *Escherichia*, *Klebsiella*, and *Enterobacter*, as defined by the modern taxonomy of the order Enterobacterales [21].
• ESBL negative: Enterobacterales isolates initially screened positive for ESBL production but failed to demonstrate positivity in confirmatory testing.
• Non‐ESBL: Enterobacterales isolates that screened negative for ESBL production were categorized as non‐ESBL.

## 6. Results

### 6.1. Demographic Characteristics of Patients

A total of 502 patients were enrolled. About 55.8% (280/502) were females and the mean age of the patients was 46 years (±20.9 SD). The larger fraction of the patients 26.7% (134/502) were in age group of 46–60 years. More than half 56.4% (283/502) of the patients were from outpatient department and majority 81.5% (409/502) of them were referred from private hospitals (Table 1).

**Table 1:** Sociodemographic characteristics and clinical characteristics of UTI suspected patients, Addis Ababa, Ethiopia from April 2025 to July 2025.

Variables		Frequency	Percentage (%)
Sex	Male	222	44.2
	Female	280	55.8
Age (years)	≤ 15	46	9.2
	16–30	80	15.9
	31–45	120	23.9
	46–60	134	26.7
	≥ 61	122	24.3
Health facility type	Private	409	81.5
	Public	93	18.5
Patient type	Outpatient	283	56.4
	Inpatient	219	43.6

### 6.2. Frequency of Enterobacterales Isolates

This study found 183 isolates out of the 502 samples from patients presented with symptoms of UTI. Of these, 179 (97.8%) were positive for bacterial growth, whereas the remaining 4 (2.2%) were attributed to fungal species. Among the bacterial isolates, 158 (88.3%) were identified as gram‐negative bacteria and 21 (11.7%) as gram‐positive bacteria. Among total urines processed, 29.4% (148/502) had a positive culture for Enterobacterales. The majority of Enterobacterales isolates were from 64.8% (96/148) female and 68.2% (101/148) outpatients. Among 148 Enterobacterales isolates, *E*. *coli* 69.6% (103/148) was the most frequent isolate followed by *K*. *pneumoniae* 16.8% (25/148). From the 103 *E*. *coli* isolates, 71.8% (74/103) were isolated from female and 72.8% (75/103) were isolated outpatient (Table 2).

**Table 2:** Distribution of Enterobacterales isolates among UTI suspected patients against demographic characteristics, health facility type and patient types, Addis Ababa, Ethiopia from April to July 2025.

Variables	Enterobacterales								Total
	*E. coli*	*K. pneumoniae*	*E. cloacae*	*C. freundii*	*K. oxytoca*	*P. mirabilis*	*M. morganii*	*S. marcascens*	
**Age (years)**									
< 15	7 (70%)	2 (20%)	0 (0%)	0 (0%)	1 (10%)	0 (0%)	0 (0%)	0 (0%)	10 (6.7%)
16–30	14 (82.3%)	2 (11.7%)	0 (0%)	0 (0%)	1 (5.8%)	0 (0%)	0 (0%)	0 (0%)	17 (11.5%)
31–45	21 (70%)	4 (13.0%)	3 (10%)	1 (3.3%)	0 (0%)	0 (0%)	0 (0%)	1 (3.3%)	30 (16.8%)
46–60	22 (61.1%)	10 (27.7%)	0 (0%)	3 (8.3%)	0 (0%)	1 (2.7%)	0 (0%)	0 (0%)	36 (24.3%)
> 61	39 (70.9%)	7 (12.7%)	3 (5.4%)	0 (0%)	2 (3.6%)	3 (5.4%)	1 (1.8%)	0 (0%)	55 (37.1%)
**Gender**									
Female	74 (77%)	12 (12.5%)	3 (3.1%)	1 (1%)	2 (2%)	0 (0%)	3 (3.1%)	1 (1%)	96 (64.8%)
Male	29 (55.7%)	13 (25%)	3 (5.7%)	3 (5.7%)	2 (3.8%)	1 (1.9%)	1 (1.9%)	0 (0%)	52 (35.2%)
**Health facility type**									
Private	96 (70%)	22 (16%)	5 (3.6	4 (2.9%)	4 (2.9%)	4 (2.9%)	1 (0.7%)	1 (0.7%)	137 (92.5%)
Public	7 (63.6%)	3 (27.2%)	1 (9%)	0 (0%)	0 (0%)	0 (0%)	0 (0%)	0 (0%)	11 (7.5%)
**Patient status**									
Outpatient	75 (74.2%)	12 (11.8%)	2 (19%)	4 (3.9%)	4 (3.9%)	3 (2.9%)	1 (0.9%)	0 (0%)	101 (68.2%)
Inpatient	28 (59.5%)	13 (27.6%)	4 (8.5%)	0 (0%)	0 (0%)	1 (2.1%)	0 (0%)	1 (2.1%)	47 (31.8%)

### 6.3. Antibiotics Resistance Pattern of Enterobacterales

In this study, among the antibiotics tested, ampicillin (98.6%) showed higher resistance rate, followed by amoxicillin‐clavulanic acid (92.5%), trimethoprim‐sulfamethoxazole (86.4%), ciprofloxacin (77.0%), cefotaxime (71.6%), ceftazidime (65.5%), and cefepime (58.1%). In contrast, meropenem (12.8%), amikacin (25%), and nitrofurantoin (36.4%) had lower resistance rates. Out of 148 Enterobacterales isolates, 122 (82.4%) were MDR, and 12 (8.1%) showed resistance to all tested antibiotics. Among the tested antibiotics, *K. pneumoniae* exhibited the highest resistance to cefotaxime (92.0%), cefepime (88.0%), and ceftazidime (92.0%). High resistance was also noted against amoxicillin‐clavulanic acid (100%) and gentamicin (80.0%), although it showed relatively lower resistance rates to meropenem (56.0%) and amikacin (56.0%). In contrast, *E. coli* showed resistance, to sulfamethoxazole‐trimethoprim (83.4%), followed by amoxicillin‐clavulanic acid (89.3%) and ciprofloxacin (72.8%). Resistance to third‐ and fourth‐generation cephalosporins was also substantial—70.8% to cefotaxime, 53.3% to cefepime, and 63.1% to ceftazidime. The most effective antibiotics against *E. coli* were meropenem and amikacin, with resistance of 2.9% and 17.4%, respectively (Table 3).

**Table 3:** Distribution of Antimicrobial resistance profile of Enterobacterales isolates.

Isolates (number)	Distribution of antibiotics resistance among Enterobacterales isolates (*n* (%))										
	AMP	AMC	CAZ	CTX	FEP	NI	AK	GEN	CIP	SXT	MER
*E. coli* (*n* = 103)	101 (98.0%)	92 (89.3%)	65 (66.0%)	73 (60.8%)	55 (53.3%)	26 (25.2%)	18 (17.4%)	32 (31.0%)	75 (72.8%)	86 (83.4%)	3 (2.9%)
*K. pneumoniae* (*n* = 25)	25 (100%)	25 (100%)	23 (92.0%)	23 (92.0%)	22 (88.0%)	18 (81.8%)	14 (56.0%)	20 (80.0%)	23 (92.0%)	23 (92.0%)	14 (56.4%)
*E. cloacae* (*n* = 6)	6 (100%)	6 (100%)	2 (33.3%)	2 (33.3%)	2 (33.3%)	2 (33.3%)	2 (33.3%)	2 (33.3%)	4 (66.6%)	5(83.3%)	2 (33.3%)
*C. freundii* (*n* = 4)	4 (100%)	4 (100%)	1 (25.0%)	1 (25.0%)	2 (50.0%)	1 (25.0%)	0 (0.0%)	0 (0.0%)	4 (100%)	0 (0.0%)	0 (0.0%)
*K. oxytoca* (*n* = 4)	4 (100%)	4 (100%)	3 (75.0%)	4 (100%)	3 (75.0%)	3 (75.0%)	2 (50.0%)	3 (75.0%)	3 (75.0%)	4 (100%)	0 (0.0%)
*P. mirabilis* (*n* = 4)	4 (100%)	4 (100%)	2 (50.0%)	2 (50.0%)	1 (25.0%)	4 (100%)	1 (25.0%)	2 (50.0%)	3 (75.0%)	4 (100%)	0 (0.0%)
*M. morganii* (*n* = 1)	1 (100%)	1 (100%)	1 (100%)	1 (100%)	1 (100%)	1 (100%)	0 (0.0%)	1 (100%)	1 (100%)	1 (100%)	0 (0.0%)
*S. marcasecens* (*n* = 1)	1 (100%)	1 (100%)	0 (0.0%)	0 (0.0%)	0 (0.0%)	1 (100%)	0 (0.0%)	1 (100%)	1 (100%)	1 (100%)	0 (0.0%)
Total resistance (*N* = 148)	146 (98.6%)	137 (92.5%)	97 (65.5%)	106 (71.6%)	86 (58.1%)	54 (36.4%)	37 (25.0%)	61 (41.2%)	114 (77.0%)	128 (86.4%)	19 (12.8%)

### 6.4. MDR Enterobacterales

Overall, 82.4% (122/148:95% CI 76.3%–88.6%) of the Enterobacterales isolates were MDR. Among this *E. coli* 54.7% (81/148) and *K. pneumoniae* 16.2% (24/148) contributed to the observed MDR. The highest MDR level were observed in *Klebsiella oxytoca*, *Citrobacter freundii*, and *Morganella morganii*, with all isolates (100%), followed by *K. pneumoniae* (96.0%, 24/25), *E. coli* (78.6%, 81/103), *Proteus mirabilis* (75%, 3/4), and *Enterobacter cloacae* (50.0%, 2/4). *S. marcescens* was found to be non‐MDR. Only 1.4% (2/148) of the Enterobacterales were susceptible for all antibiotics tested (Table 3). From all MDR Enterobacterales, the predominant were *E. coli* (54.7%; 81/148) and *K. pneumoniae* (16.2%, 24/148) (Table 4).

**Table 4:** Multidrug resistance level of Enterobacterales to different classes of antibiotics from April to July 2025.

Isolates (number)	Level of antibiotics resistance (number (%))								MDR‐E (>R3)
	R0	R1	R2	R3	R4	R5	R6	R7	
*E. coli* (103)	2 (1.9)	11 (10.7)	9 (8.7)	14 (13.5)	41 (39.8)	18 (17.4)	7 (6.7)	1 (0.9)	81 (78.6)
*K. pneumoniae* (25)	0 (0.0)	1 (4.0)	0 (0.0)	2 (8.0)	2 (8.0)	4 (16.0)	7 (28.0)	9 (36.0)	24 (96.0)
*E. cloacae* (4)	0 (0.0)	1 (25.0)	1 (25.0)	2 (50.0)	0 (0.0)	0 (0.0)	0 (0.0)	2 (50.0)	2 (50.0)
*C. freundii* (4)	0 (0.0)	0 (0.0)	0 (0.0)	2 (50.0)	2 (50.0)	0 (0.0)	0 (0.0)	2 (50.0)	4 (100.0)
*K. oxytoca* (4)	0 (0.0)	0 (0.0)	0 (0.0)	1 (25.0)	0 (0.0)	2 (50.0)	1 (25.0)	0 (0.0)	4 (100.0)
*P. mirabilis (4)*	0 (0.0)	0 (0.0)	1 (25.0)	0 (0.0)	1 (25.0)	1 (25.0)	1 (25.0)	0 (0.0)	3 (75.0)
*M. morganii* (1)	0 (0.0)	0 (0.0)	0 (0.0)	0 (0.0)	0 (0.0)	1 (100.0)	0 (0.0)	0 (0.0)	1 (100.0)
*S. marcascens* (1)	0 (0.0)	0 (0.0)	0 (0.0)	0 (0.0)	1 (100)	0 (0.0)	0 (0.0)	0 (0.0)	0 (0.0)
Total (*N* = 148)	2 (1.3)	13 (8.7)	11 (7.4)	21 (14.1)	47 (31.7)	26 (17.5)	16 (10.8)	12 (8.1)	122 (82.4)

Abbreviations: R0,Absence of resistance to any antibiotic category; R1, resistance to one antibiotic category; R2,resistance to two antibiotic categories; R3, resistance to three antibiotic categories; R4, resistance to four antibiotic categories; R5, resistance to five antibiotic categories; R6, resistance to six antibiotic categories and R7, resistance to seven antibiotic categories used for treatment.

**Figure 1:**
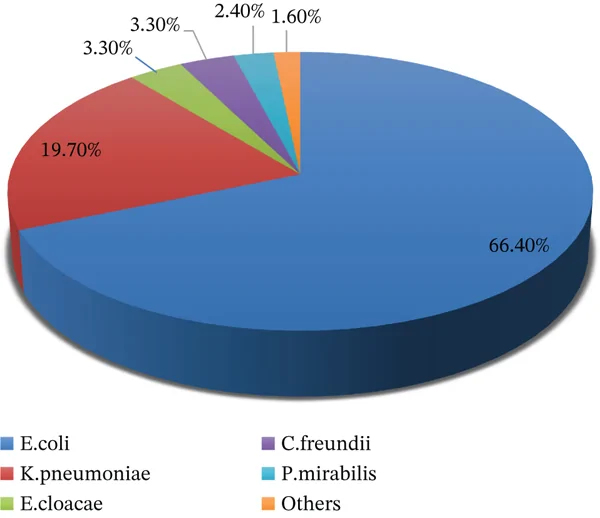
Distribution of major MDR isolate among total MDR Enterobacterales in this study. Note: Others are *S. marcescens* and *M. morganii.*

Out of all 122 MDR Enterobacterales isolates, the most frequently identified species was *E. coli* (66.4%, 81/122), followed by *K. pneumoniae* (19.7%, 24/122). In addition, *C. freundii* (3.3%, 4/122), *E. cloacae* (3.3%, 4/122), *K. oxytoca* (3.3%, 4/122), and *P. mirabilis* (2.4%, 3/122) were the remaining isolates (Figure 1).

### 6.5. MAR Index

The MAR index was calculated for all 148 Enterobacterales isolates. The overall mean MAR index was 0.605. A total of 132 (89.2%) isolates had a MAR index > 0.20, whereas only 16 (10.8%) had a MAR index ≤ 0.20.

Among the bacterial species, *K. pneumoniae* exhibited the highest mean MAR index (0.836), followed by *M. morganii* (0.820). In contrast, *C. freundii* had the lowest mean MAR index (0.477), followed by *E. cloacae* (0.530). Overall, the high MAR index observed across most bacterial species suggests that these isolates likely originated from environments with considerable antibiotic selective pressure (Table 5).

**Table 5:** Distribution of bacterial isolates and their corresponding mean multiple antibiotic resistance (MAR) index.

Isolates	Number of isolates	Mean MAR Index
*K. pneumoniae*	25	0.836
*M. morganii*	1	0.820
*P. mirabilis*	4	0.568
*E. coli*	103	0.553
*S. marcescens*	1	0.550
*E. cloacae*	6	0.530
*C. freundii*	4	0.477

Abbreviation: MAR, multiple antibiotic resistance index.

### 6.6. Magnitude of ESBL‐E

Among Enterobacterales isolates, 72.3% (107/148) were positive for the screening test of ESBL production. Of the 107 screening positive isolates, 97 (90.6%) were confirmed ESBL producers by the CDT, resulting in an overall ESBLs positivity of 65.5% (97/148: 95% CI: 57.8%–73.2%) (Table 6).

**Table 6:** Distribution of major ESBL‐producing Enterobacterales and MDR isolates among inpatients and outpatients presenting symptoms of UTI at WAML, Addis Ababa, Ethiopia, April–July 2025.

Isolate collection Site	ESBL‐producing Enterobacterales (*n* (%))	Major ESBL‐producing Enterobacterales			MDR Enterobacterales (*n* (%))
		*E. coli* (*n* (%))	*K. pneumoniae* (*n* (%))	*E. coli* and *K. pneumoniae* (*n* (%))	
Outpatient	64.3% (65/101)	61.3% (46/75)	91.6% (11/12)	65.5% (57/87)	77.2% (78/101)
Inpatient	68.0% (32/47)	64.2% (18/28)	92.3% (12/13)	73.1% (30/41)	93.6% (44/47)
Total	65.5% (97/148)	62.1% (64/103)	92.0% (23/25)	67.9% (87/128)	82.4% (122/148)

**Figure 2:**
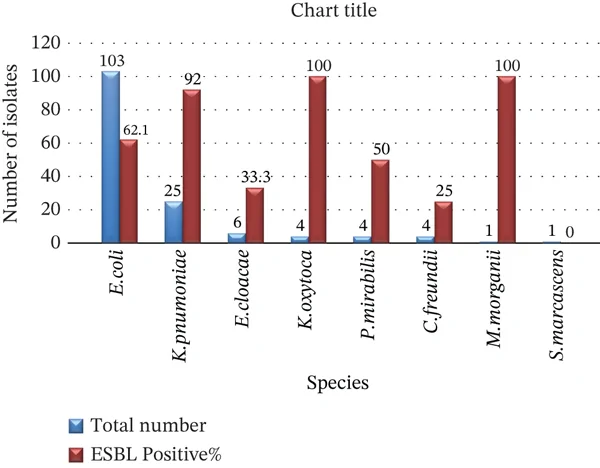
Frequency of ESBL producing Enterobacterales among UTI patients at WAML from April to July 2025.

ESBL production showed notable variation across Enterobacterales species. *K. oxytoca* (4/4) and *M. morganii* (1/1) exhibited the highest intraspecies ESBL production rates, with both showing 100% positivity. This was followed closely by *K. pneumoniae*, where 92.0% (23/25) of isolates were ESBL producers. Moderate levels of ESBL production were seen in *E. coli* and *P. mirabilis*, with 62.1% (64/103) and 50% (2/4), respectively. On the other hand, lower frequencies were observed among *E. cloacae* and *Citrobacter* species, with 33.3% (2/6) and 25% (1/4) ESBL‐positive isolates, respectively (Figure 2). A significantly higher proportion of MDR was found among ESBL‐E (95.8%; 93/97) than in non–ESBL‐producing isolates (54.9%; 28/51) (Figure 2).

Higher frequencies of both ESBL and MDR were observed among older patients, particularly those aged above 61 years. ESBL production was slightly more common among females, whereas MDR prevalence was higher among males. Inpatients exhibited a greater proportion of MDR isolates compared with outpatients. *E. coli* and *K. pneumoniae* were the predominant bacterial species and accounted for the majority of both ESBL producing and MDR isolates. (Table 7).

**Table 7:** Distribution of ESBL production and multidrug resistance (MDR) according to demographic characteristics, healthcare facility type, ward, and bacterial isolate.

Variable	Category	ESBL positive *n* (%)	ESBL negative *n* (%)	MDR *n* (%)	Non‐MDR *n* (%)
Age (years)	< 15	7 (100.0)	0 (0.0)	6 (60.0)	4 (40.0)
	16–30	10 (100.0)	0 (0.0)	12 (70.6)	5 (29.4)
	31–45	16 (88.9)	2 (11.1)	24 (80.0)	6 (20.0)
	46–60	26 (96.3)	1 (3.7)	32 (88.9)	4 (11.1)
	> 61	38 (84.4)	7 (15.6)	48 (87.3)	7 (12.7)
Sex	Male	38 (88.4)	5 (11.6)	45 (86.5)	7 (13.5)
	Female	59 (92.2)	5 (7.8)	77 (80.2)	19 (19.8)
Healthcare facility type	Private	91 (90.1)	10 (9.9)	116 (84.7)	21 (15.3)
	Public	6 (100.0)	0 (0.0)	6 (54.5)	5 (45.5)
Ward	Outpatient	65 (89.0)	8 (11.0)	78 (77.2)	23 (22.8)
	Inpatient	32 (94.1)	2 (5.9)	44 (93.6)	3 (6.4)
Bacterial isolate	*C. freundii*	1 (50.0)	1 (50.0)	4 (100.0)	0 (0.0)
	*E. cloacae*	2 (100.0)	0 (0.0)	4 (66.7)	2 (33.3)
	*E. coli*	64 (87.7)	9 (12.3)	81 (78.6)	22 (21.4)
	*K. oxytoca*	4 (100.0)	0 (0.0)	4 (100.0)	0 (0.0)
	*K. pneumoniae*	23 (100.0)	0 (0.0)	24 (96.0)	1 (4.0)
	*M. morganii*	1 (100.0)	0 (0.0)	1 (100.0)	0 (0.0)
	*P. mirabilis*	2 (100.0)	0 (0.0)	3 (75.0)	1 (25.0)
	*S. marcescens*	—	—	1 (100.0)	0 (0.0)

### 6.7. Antibiotics Susceptibility Pattern of ESBLs‐E

**Figure 3:**
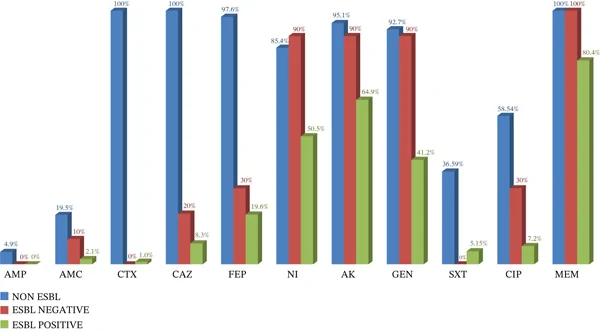
Antibiotics susceptibility pattern of ESBLs‐positive, ESBLs‐negative, and non‐ESBL from April to July 2025. Abbreviations: AMC, amoxicillin/clavulanate; AMP, ampicillin; CIP, ciprofloxacin; GEN, gentamicin; STX, trimethoprim‐sulfamethoxazole; NI, nitrofurantoin; CTX, cefotaxime; CAZ, ceftazidime; AK, amikacin; FEP, cefepime; MEM, meropenem.

Meropenem (80.4%) and amikacin (64.9%) were the most effective antibiotics against ESBL producing Enterobacterales. However, gentamicin, ciprofloxacin, and cotrimoxazole showed lower susceptibility, with 41.2%, 7.2%, and 5.2%, respectively. In contrast, non‐ESBL, screening negative bacterial isolates showed higher sensitivity to meropenem (100%), amikacin (95.1%), and gentamicin (92.7%). Additionally, nitrofurantoin and ciprofloxacin were effective against non‐ESBL isolates with sensitivity of 85.4% and 58.6%, respectively. (Figure 3).

## 7. Discussion

ESBL‐E has become a critical global concern. The spread of these enzymes significantly reduces the effectiveness of broad‐spectrum antibiotics, leading to serious treatment challenges and influencing patient outcomes.

In this study, the prevalence of ESBL‐E was 65.5%, which aligns closely with findings from Ethiopia (67.0%) [22], Tanzania (64%) [23], Cameroon (64.7%) [24], and Uganda (64.9%) [25]. Compared to a previous study, the prevalence of ESBL production found in the current study is notably lower than from a study in Bahir Dar, Ethiopia (85.8%) [26]. The ESBL prevalence was also lower than those reported in Egypt (70%) [27], this high prevalence might be attributed to the fact that the study was conducted on clinical samples, which are more likely to yield resistant strains.

On the contrary, this study reported a higher prevalence of ESBL‐producing isolates compared to other findings from Addis Ababa (57.7%) [4], Adama (25%) [28], Chad (47.7%) [29], South Africa (59.0%) [30], and Sudan (45.2%) [31], suggesting a rising trend in the spread of ESBL‐E across Africa, which may be attributed to the frequent empirical use of third‐generation cephalosporin′s, However, lower prevalence rates have been reported in developed regions like the United States, Europe [32], Australia [33], and several Asian countries [34, 35]; this may be attributed to variations in infection prevention protocols, duration of hospitalization, and enhanced nursing practices that effectively reduce the transmission and acquisition of ESBL‐producing strains.

Similar to previous findings, *E. coli* remained the predominant uropathogen, with *K. pneumoniae* being the second most common [22]. Although *E. coli* was the most commonly isolated organism (69.5%), *K. pneumoniae* exhibited the highest rate of ESBL production (92.0%), with *E. coli* at 62.1%. A similar trend was observed in studies conducted in Ethiopia [36, 37]. However, other studies from Ethiopia reported *E. coli* as the predominant ESBL producer compared to *K. pneumoniae* with rates of 58% for *E. coli* and 23% for *K. pneumoniae* [15]. Comparable findings were also reported in Cameroon (*E. coli* 79.4% and *K. pneumoniae* 14.7%) [24] and Iraq (*E. coli* 71.7% and *K. pneumoniae* 26.7%) [38].

Although the overall prevalence of ESBL‐E was 65.5%, analysis by patient type showed a slightly higher prevalence among inpatients (68.0%) than outpatients (64.3%). Similarly, MDR was more common among inpatient isolates (93.6%) than outpatient isolates (77.2%). These findings suggest that hospitalized patients remain an important reservoir of resistant Enterobacterales, likely due to prolonged hospitalization, greater exposure to broad‐spectrum antibiotics, invasive procedures such as urinary catheterization, and underlying comorbidities. Nevertheless, the high prevalence of ESBL‐producing isolates among outpatients indicates that these resistant organisms are also well established in the community, highlighting that combining inpatient and outpatient data may underestimate important epidemiological differences between these populations. Similar observations have been reported in studies from Ghana and Rwanda [39, 40].

In this study, the highest level of resistance was observed against ampicillin (98.6%), followed by amoxicillin‐clavulanic acid (92.5%), sulfamethoxazole‐trimethoprim (86.4%), ciprofloxacin (77.0%), cefotaxime (71.6%), ceftazidime (65.5%), and cefepime (58.1%). These findings are consistent with previous reports from Addis Ababa, Ethiopia [4]. High levels of resistance were observed in *E. coli* against sulfamethoxazole‐trimethoprim (77.6%), amoxicillin‐clavulanic acid (70.0%), and ciprofloxacin (64.0%). Resistance to third‐ and fourth‐generation cephalosporins was also notable, with rates of 54.8% for cefotaxime, 53.5% for cefepime, and 53.1% for ceftazidime. Similar findings have been reported in earlier research carried out in Addis Ababa, Ethiopia [14], Nigeria [41], and Tanzania [42].

The high resistance observed against commonly prescribed antibiotics may reflect sustained antimicrobial selective pressure in both hospital and community settings. In Ethiopia, empirical treatment of UTIs, inappropriate antibiotic prescribing, self‐medication, over‐the‐counter access to antibiotics, and limited implementation of antimicrobial stewardship programs may contribute to the persistence and spread of resistant Enterobacterales. A high prevalence of isolates with a MAR index > 0.20 reinforces the assumption that these organisms emerged from environments characterized by heavy antibiotic usage. In contrast, the relatively preserved activity of meropenem and amikacin suggests that these agents remain effective because they are used less frequently and are generally reserved for severe infections.

In this study, MDR was detected in 93 (95.8%) of the ESBL‐producing isolates compared to only 28 (54.9%) of the non‐ESBL producers, which aligns closely with findings from Addis Ababa, Ethiopia [9]. This strong association indicates that ESBL production frequently coexists with resistance to multiple non–*β*‐lactam antimicrobial classes, substantially limiting available treatment options. The higher MDR burden observed among inpatient isolates further emphasizes the importance of strengthening infection prevention measures and antimicrobial stewardship in healthcare settings.

The current study found that ESBL‐E isolates were most susceptible to meropenem (80.4%), followed by amikacin (64.9%) and nitrofurantoin (50.5%). These results align with previous findings from Addis Ababa, Ethiopia [14] and Nigeria [41]. This suggests meropenem, amikacin, and nitrofurantoin appear to remain effective choices for treating infections due to ESBL‐E.

## 8. Strengths and Limitations

A major strength of this study was the use of MALDI‐TOF for accurate bacterial identification. Additionally, the study included a sample size that exceeded the calculated minimum required sample size, thereby enhancing the robustness and reliability of the findings.

However, several limitations should be considered. Molecular characterization of ESBL genes was not performed. Important clinical risk factors such as prior hospitalization, urinary catheterization, recurrent UTI, and urinary tract abnormalities were not assessed. Convenience sampling may have introduced selection bias.

## 9. Conclusion

This study found a high prevalence of ESBL‐E and MDR Enterobacterales among patients suspected of UTIs, particularly among hospitalized patients and those attending private hospitals. *K. pneumoniae* and *E. coli* were the most frequently identified ESBL‐producing bacteria. ESBL‐producing isolates exhibited higher resistance rates to commonly used antibiotics, including trimethoprim‐sulfamethoxazole, ciprofloxacin, cefepime, and gentamicin, compared with non–ESBL‐producing isolates. In contrast, meropenem, amikacin, and nitrofurantoin demonstrated comparatively better activity against ESBL‐E isolates.

## 10. Recommendation

The increasing prevalence of MDR organisms and ESBLs highlights the urgent need to advance clinical bacteriology research and enhance laboratory diagnostic capacities for accurate detection and surveillance of antimicrobial resistance. Therefore, routine screening of ESBL‐E, along with strong infection prevention and control measures, is needed. In addition, raising awareness about antimicrobial resistance through comprehensive education programs, enforcing rigorous antimicrobial stewardship practices, and promoting the judicious use of antibiotics by healthcare providers are essential. Moreover, researchers should focus on identifying the dominant ESBL genes circulating in Ethiopia to gain deeper insights into local resistance patterns and mechanisms.

**Nomenclature**
AR: antibiotic resistance
ATCC: American Type Culture Collection
CLSI: Clinical Laboratory Standards Institute
CDT: combined disc test
ESBL: extended spectrum beta‐lactamase
ESBL‐E: extended spectrum *β*‐lactamase–producing Enterobacterales
MALDI‐TOF: matrix‐assisted laser desorption/ionization time‐of‐flight
MDR: multidrug resistance
SOP: standard operating procedure
Spp: species
SPSS: Statistical Package for Social Sciences statistical software
UTIs: urinary tract infections
WAML: Wudassie Advanced Medical Laboratory
WDC: Wudassie Diagnostic Center

## Author Contributions

Tamirat Tamiru conceptualized the study, designed the methodology, collected and analyzed the data, interpreted the findings, and drafted the manuscript. Kassu Desta, Regasa Diriba, and Tadesse Shume supervised the study through their critical review of the research and the manuscript. Yordanos Getachew, Mekdelawit Fisseha, and Sami Sebri participated in the technical laboratory works and data collection.

## Funding

No funding was received for this manuscript.

## Disclosure

All authors have read and approved the final manuscript.

## Ethics Statement

The study was conducted in accordance with the ethical principles of the Declaration of Helsinki. Ethical approval for this study was obtained from the Departmental Research and Ethics Review Committee (DRERC), Department of Medical Laboratory Sciences, College of Health Sciences, Addis Ababa University, Addis Ababa, Ethiopia (Ref.No. DRERC/783/24/MLS/).

## Consent

Participants were told of the purpose of the study, and written informed consent was taken from each patient, and informed assent was taken from their parents or guardians on behalf of children and ICU patients before data collection. Each respondent was given the right to refuse to take part in the study and to withdraw at any time during the study period. All the information obtained from the study subjects was coded to maintain confidentiality.

## Conflicts of Interest

The authors declare no conflicts of interest.

## Data Availability

The data that support the findings of this study are available from the corresponding author upon reasonable request.
